# Painful Hip Leading to the Diagnosis of MEN 2B Syndrome

**DOI:** 10.1155/2012/567060

**Published:** 2012-11-26

**Authors:** Mehtab Ahmad, Imran Rizvi, Amit Jain, Noorin Zaidi

**Affiliations:** ^1^Department of Radiodiagnosis, J.N. Medical College, Aligarh 202002, India; ^2^Department of General Medicine, J.N. Medical College, Aligarh 202002, India; ^3^Department of Pathology, J.N. Medical College, Aligarh 202002, India

## Abstract

*Context*. MEN 2B syndrome is characterized by the presence of medullary thyroid cancer, pheochromocytoma, mucosal neuromas, marfanoid features, and skeletal abnormalities like kyphoscoliosis, joint laxity, pes cavus, and slipped capital femoral epiphysis (SCFE) in a minority; we present the case of a young female who was brought to medical attention due to painful hip because of SCFE. *Case Report*. A 16-year-old female presented to orthopedics out-patient department (OPD) with complaints of pain around the left hip and walking with a limp for the last two months. MRI of hip confirmed the presence of SCFE of the left hip. General examination detected thyroid swelling which was found to be a medullary thyroid cancer and imaging of abdomen confirmed the presence of bilateral pheochromocytoma, also present were neuromas of tongue and lips. Thus, a diagnosis of MEN 2B syndrome was made. *Conclusion*. SCFE can sometimes be the presenting feature of MEN 2B syndrome. Physicians should keep this in mind as it can lead to early diagnosis of a potentially lethal illness.

## 1. Introduction

Multiple endocrine neoplasia is a rare, hereditary, neoplastic syndrome with autosomal dominant inheritance [1]. It is characterized by the presence of multiple hormones producing tumors in specific endocrine organs and is classified into three types: (i) MEN 1, which comprises of pituitary adenomas, primary hyperparathyroidism (PHPT), and pancreatic islet cell tumors; (ii) MEN 2A, which includes medullary thyroid carcinoma (MTC), pheochromocytoma, and PHPT; (iii) MEN 2B, which is rarer and more aggressive type of MEN 2 and comprises for 5–10% of all. It consists of MTC, pheochromocytoma, ganglioneuromas, physical features such as presence of Marfanoid habitus, raised bumps on the lips, tongue, and eyelids (due to cutaneous neuromas), thickened corneal nerves (seen on slit lamp examination), and skeletal abnormalities (kyphoscoliosis, joint laxity, pes cavus, and slipped capital femoral epiphysis in a minority) [2]. Usual presentation of the syndrome is hypertension or thyroid nodule, we present the case of a young female who was brought to medical attention due to painful hip but further workup proved her to be suffering from MEN 2B syndrome.

## 2. Case History

**Figure 1:**
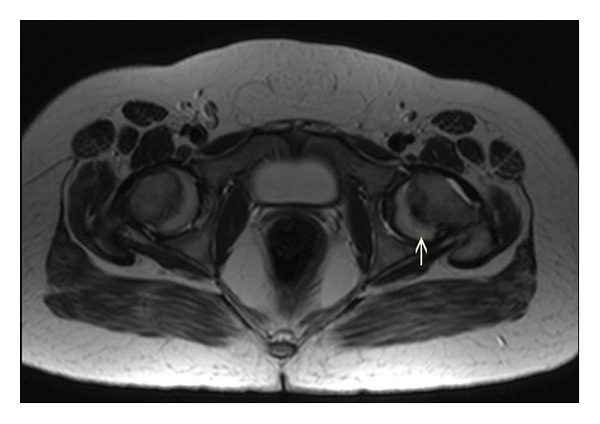
Axial T2-weighted MR image at the level of bilateral hip joint showing posterior displacement of the epiphysis of femoral head on left side (arrow).

A 16-year-old female presented to orthopedics out-patient department (OPD) with complaints of pain around the left hip and walking with a limp for the last two months. Clinical examination showed left hip to be in the state of flexion, abduction, and external rotation, and there was decreased range of motion. X-ray and subsequent MRI confirmed a slipped capital femoral epiphysis (SCFE) of the left hip (Figure 1). On general examination, an irregular bumpy swelling was noted in the anterior neck. On enquiry, patient said that she had this swelling for about 6 months and was progressively increasing; she had consulted some local quack for the neck swelling who diagnosed it as endemic goiter. 

Her pulse rate was 106/minute and blood pressure was 174/80 mm Hg. She was referred to medicine OPD for hypertension where her complete evaluation was done. On examination in the medicine clinic, she was found to have multiple neuromas on the oral mucosa, lips, and tongue.

Height of the patient was 164 cm and arm span was 168 cm, upper-to-lower segment ratio was 0.77. She had long, thin arms and legs with arm span more than her height suggesting Marfanoid habitus. Weight of the patient was 41 kg.

Laboratory findings showed normal haematological profile, her blood sugar, blood urea, serum creatinine, and urine routine examination; TSH, T3, and T4 were all within normal range. Urinary metanephrines and vanillyl mandelic acid (VMA) were found to be elevated in 24-hour urine sample (metanephrines 4.2 mg and VMA 24.1 mg in 24 hour urinary sample). Serum levels of calcitonin were also found to be elevated.

**Figure 2:**
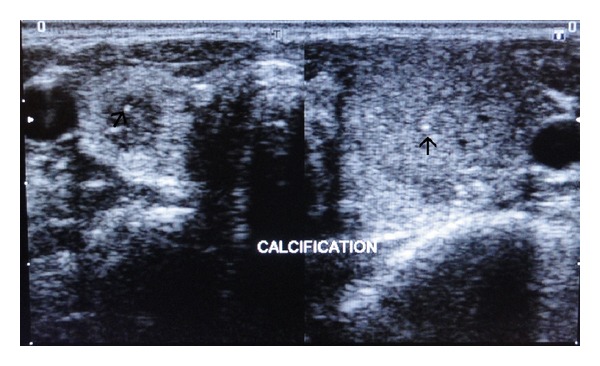
Axial ultrasound images taken just above the level of thyroid isthmus showing a well-defined, heterogeneous, predominantly hypoechoic round lesion in the right lobe of thyroid. Small echogenicities within the lesion were noted suggestive of microcalcification (black arrows). Another larger heterogeneous solid lesion is seen in the left lobe of thyroid.

**Figure 3:**
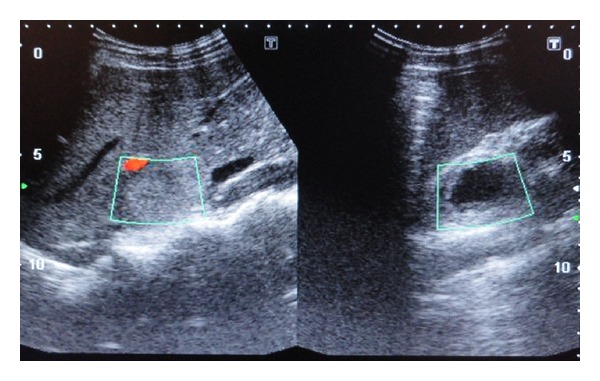
Ultrasound of abdomen showing heterogeneous lesions at the upper poles of both kidneys suggestive of bilateral adrenal masses.

**Figure 4:**
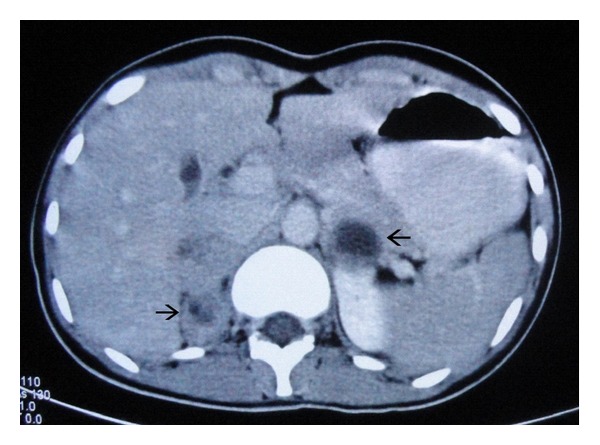
Axial contrast enhanced CT images of abdomen taken at the level of adrenals showing large round heterogeneous lesions in bilateral adrenals with left adrenal lesion showing central necrosis and fluid-fluid level. Central hypodense nonenhancing area is suggestive of necrosis with intense peripheral enhancement.

Ultrasonography of the anterior neck showed well-defined, heterogeneous, predominantly hypoechoic round lesions in both the lobes of thyroid measuring 22.4 × 17.6 mm on left side and 10 × 6.9 mm on the right with tiny echogenic foci consistent with calcification within the lesion (Figure 2). Fine needle aspiration cytology (FNAC) was consistent with medullary thyroid carcinoma. Ultrasound screening of abdomen showed heterogeneous hypoechoic lesions at the upper poles of bilateral kidneys consistent with bilateral adrenal masses (Figure 3). Abdominal computed tomography (CT) scan showed large, bilateral, heterogeneous suprarenal lesions with central necrosis and intense peripheral postcontrast enhancement consistent with pheochromocytomas (Figure 4). No evidence of bowel ganglioneuromas was seen on CT scan, also Slit lamp examination by ophthalmologist did not reveal any thickened corneal nerve. There was no family history of thyroid nodules, mucosal neuromas, or hypertension. Genetic analysis could not be performed because the facility of the same was not available at our centre. On the basis of MTC, pheochromocytoma, neuroma of tongue, and Marfanoid habitus, she was diagnosed as a case of multiple endocrine neoplasia type 2B. The patient was referred to the nearest oncologic facility for expert management.

## 3. Discussion

MEN 2 was first described in 1961 [3]. Since then, detailed descriptions of clinical syndromes associated with MEN 2 have followed. MEN 2B is also called Wagenmann-Froboese or mucosal neuroma syndrome. It accounts for 5–10% of cases of MEN 2 [2]. The disease is transmitted as autosomal dominant, but a large proportion of the cases does not have any family history and arise as a result of *de novo* mutation.

In 1993, germline missense mutation was found in RET protooncogene on chromosome 10q11.2, that is, which is a point mutation in the methionine residue in exon 16 and is responsible for about 95% of MEN 2B cases [4]. 

Approximate mean age for the diagnosis of MEN 2B syndrome is 11.5 years [5]. The Most common manifestation of this disease is the occurrence of mucocutaneous neuromas especially on the tongue and subconjunctival areas. The MTC is the next common component of MEN 2B, which can have an onset as early as first year of life [6]. In contrast to MEN 2A in which MTC has an indolent course in 80% of cases, in MEN 2B MTC has a very aggressive course and is rarely curable. These hereditary MTCS are typically bilateral and metastasize early. As in our case, these tumours are located at the junction of upper and middle third of thyroid lobes coherent with the maximum density of c-cells in this area.

Pheochromocytoma is another very important component which occurs in about half of patients with MEN 2B [7, 8]. The usual age of presentation is around 30 years. Approximately, 50% of the tumours are bilateral as in the case under discussion. The complete syndrome with mucosal neuromas, pheochromocytoma, and MTC occurs in only 50% of the cases [5]. Generally, pheochromocytoma is the first clinical manifestation of the disease in 25% of cases, MTC in 40%, and in 35% of cases MTC and pheochromocytoma are diagnosed at the same time.

Slipped capital femoral epiphysis as a presenting feature of MEN 2B has been very rarely described in the literature previously [9, 10]. Exact incidence of this association cannot be determined due to rarity.

Management of the MEN 2B is complex and demands a high level of coordination between a multidisciplinary team, involving medical, surgical, and oncologic treatment options to be applied according to the requirement of each individual case. The appropriate surgery for sporadic MTC and index patients in MEN 2 is total thyroidectomy and lymph node dissection of the central and, if required, lateral compartment of the neck.

Total thyroidectomy is an absolute requirement in all cases of MEN 2B because of the bilateral and multifocal nature of MTC. Lifelong L-Thyroxine replacement therapy should be started in all patients after total thyroidectomy. All postoperative patients should be followed with regular calcitonin and carcinoembryonic antigen (CEA) determination [11]. Normal basal and pentagastrin-stimulated calcitonin levels imply a tumour-free state and such patients necessitate only regular followedup at half yearly intervals with physical examination and calcitonin determination. However, if the initial surgical procedure is found to be inadequate, then reoperation with an appropriate surgical procedure is indicated [12]. 

The presence of concurrent pheochromocytoma must be ruled out prior to any surgical procedure. A coexisting pheochromocytoma should be removed before thyroidectomy. Bilateral disease of adrenal glands is common in MEN 2B patients, and because of risks associated with long-term adrenocortical supplementation therapy following adrenalectomy, adrenal cortex-sparing surgery is recommended [13].
